# Response rate of patient reported outcomes: the delivery method matters

**DOI:** 10.1186/s12874-021-01419-2

**Published:** 2021-10-22

**Authors:** Olaf M. Neve, Peter Paul G. van Benthem, Anne M. Stiggelbout, Erik F. Hensen

**Affiliations:** 1 Department of Otorhinolaryngology and Head & Neck Surgery, Leiden University Medical Center, P.O. Box 9600, 2300 RC Leiden, Zuid-Holland The Netherlands; 2 Medical Decision Making, Department of Biomedical Data Sciences, Leiden University Medical Center, Leiden, Zuid-Holland The Netherlands

**Keywords:** Response rate, Delivery method, Patient-reported outcome, Vestibular schwannoma

## Abstract

**Background:**

Patient Reported Outcomes (PROs) are subjective outcomes of disease and/or treatment in clinical research. For effective evaluations of PROs, high response rates are crucial. This study assessed the impact of the delivery method on the patients’ response rate.

**Methods:**

A cohort of patients with a unilateral vestibular schwannoma (a condition with substantial impact on quality of life, requiring prolonged follow-up) was assigned to three delivery methods: email, regular mail, and hybrid. Patients were matched for age and time since the last visit to the outpatient clinic. The primary outcome was the response rate, determinants other than delivery mode were age, education and time since the last consultation. In addition, the effect of a second reminder by telephone was evaluated.

**Results:**

In total 602 patients participated in this study. The response rates for delivery by email, hybrid, and mail were 45, 58 and 60%, respectively. The response rates increased after a reminder by telephone to 62, 67 and 64%, respectively. A lower response rate was associated with lower level of education and longer time interval since last outpatient clinic visit.

**Conclusion:**

The response rate for PRO varies by delivery method. PRO surveys by regular mail yield the highest response rate, followed by hybrid and email delivery methods. Hybrid delivery combines good response rates with the ease of digitally returned questionnaires.

## Background

Patient Reported Outcomes (PROs) are increasingly used both for scientific purposes and in clinical practice. PROs measure the patients’ perceived symptoms, functioning, and health-related quality of life. The use of PROs in research improves understanding the patient’s perspective on the disease, the sequelae, and therapy [1]. In addition, using PROs in clinical practice may improve patient-clinician communication and enhance patient outcomes [2, 3]. However, the implementation of PROs in routine practice can be challenging due to technological and workflow barriers [2].

One such barrier can be the response rate. A low response rate can lead to the introduction of selection bias and reduce the outcomes’ external validity [4]. In general, response rates can be improved by several methods including monetary incentives, shorter questionnaires, reminders, personally addressed invitations and delivery method [5–8]. Delivery by email is increasingly used, with both distribution and digital data entry of the answers saving costs. However, delivery by regular mail has seemed to provide better response rates over the years [8]. Research performed in the medical context has shown that clinicians’ response rates are similar or slightly in favor of mail delivery compared to email [9, 10]. A hybrid delivery method using both mail and email might be better than either email or mail alone [11]. Research on delivery method and patients’ response rates is scarce and often performed in small sample sizes. These studies, published between 2014 and 2017, have shown that mail delivery results in higher response rates compared to email delivery [12–14]. However, digital literacy has rapidly increased in recent years. For example, in Europe 87% of the people aged 16–74 years had used internet in the last 3 months in 2019 compared to 75% in 2013, and 57% in 2007 [15]. As a result, patients’ response to email may have increased too. This study assessed three different delivery methods for PRO measures in a large cohort of patients with unilateral vestibular schwannoma.

## Methods

This study was part of a larger study on long-term outcomes of vestibular schwannoma management. Vestibular schwannoma is a benign, usually not life-threatening intracranial tumor, causing symptoms such as hearing loss, tinnitus, and balance problems due to pressure on adjacent structures, and as such may have considerable impact on quality of life. A small majority of these tumors is non-progressive and in these cases active surveillance during an extended follow-up period is usually the management option of choice. In progressive tumors, surgery or radiotherapy is performed to prevent future complications such as brain stem compression or elevated intracranial pressure. After an active intervention, prolonged active surveillance ensues in these patients too, in order to identify possible recurrences.

Patients who participated in a survey study in 2014 were re-approached for participation in a survey between May and September 2020 [16]. Both studies were performed at the Leiden University Medical Center, an expert referral center for vestibular schwannoma in the Netherlands. All patients were diagnosed with unilateral VS between 2003 and 2014. Patients with bilateral VS, other skull base pathologies or insufficient proficiency in the Dutch language to complete the questionnaires were excluded.

Several PRO measures that are used in the routine care for vestibular schwannoma care in our hospital were collected in this study. Patients received a general health-related quality of life (HRQL) questionnaire, the short form 36 (SF-36), and a disease-specific HRQL questionnaire, the Penn Acoustic Neuroma Quality-of-Life Scale (PANQOL) [17, 18]. In addition, patients were asked to complete the dizziness handicap inventory (DHI), the medical outcome study cognitive functioning scale (MOS-CFS), the decision regret scale and the productivity costs questionnaires (iPCQ) [19–21]. Combined, patients were asked to answer 117 questions.

Three different delivery methods were used: email, regular mail, and a hybrid of the two. These three methods were chosen because they represented the modern delivery method (email), the golden standard so far (mail) and an intermediate (hybrid) method that combines the conventional approach of mail with the advantage of digital data entry. Patients in the email group received an email invitation with a link to a digital informed consent form. After providing consent, patients were directed to digital questionnaires. Patients in the hybrid group were invited by regular mail with a letter including a unique code and a link to the digital informed consent form and the questionnaires. The regular mail group received an informed consent form, the printed questionnaires, and a pre-paid return envelope. After 2 weeks, patients received a first personally addressed reminder by email (email group) or mail (hybrid and regular mail group). After another 2 weeks, all non-responders were called once by telephone for a second reminder. This telephone call was performed by a researcher, not their treating physician. In all groups, patients could request a different delivery method. Responders were defined as patients who completed the informed consent form and opened the questionnaire.

**Fig. 1:**
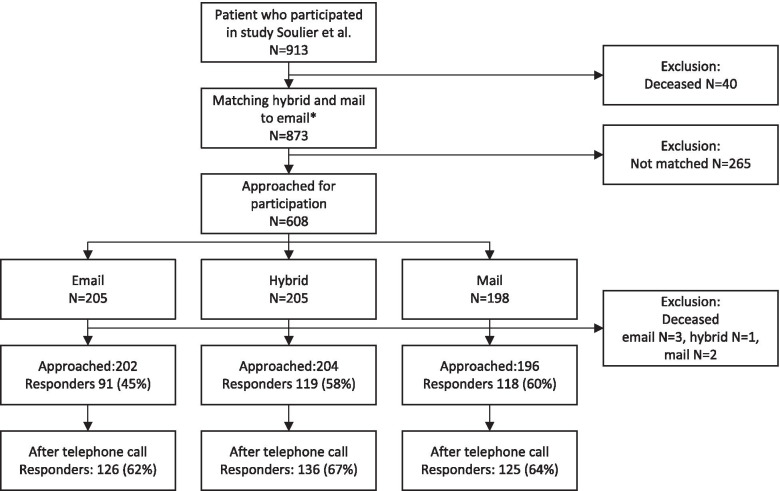
Flowchart of study participants. Patients who participated in a previous study in 2014 were reapproached for participation. Before 2011 email was not registered at the first visit to the hospital. As a result, an email was available for a minority of the patients making randomization impossible. * All groups were matched for age and the time since the last visit

Before introducing electronic patient records in 2011, the patients’ email address was not registered during the first visit to the hospital. Therefore, an email address was available for a minority of the patients, making randomization impossible. Patients for whom the email address was registered were assigned to the email group. Patients from whom no email address was available were randomly assigned to either the regular mail or hybrid delivery groups. Two factors, age and time since the last visit, were expected to differ between groups with and without email, since most patients without email addresses were diagnosed before 2011. To avoid confounding of the effect of the delivery method on the response rate by two factors, we matched patients in all groups for age (< 45 yrs.; 46-50 yrs.;…;81-85 yrs.;> 85 yrs) and time since the last visit (< 5 yrs.;5-10 yrs.;> 10 yrs), as is shown in Fig. 1.

The frequencies of categorical variables and means of numerical variables were calculated. Demographics of responders and non-responders were compared. Next, three analyses were performed because patients could switch delivery methods. First, a stringent analysis was performed in which switchers were considered as non-responders. Second, an intention to treat analysis was conducted in which patients were analyzed in their predefined delivery method. Third, an as treated analysis was performed in which patients who switched between delivery methods were analyzed in that category. The outcome was the response rate per group, which was analyzed using a chi-squared test. We also assessed the effect of the second telephone call reminder by a chi-squared test. In addition, the effect on the response rate of age, sex, education level, the time elapsed since the last visit (in years), and the delivery method were analyzed using logistic regression with response rate as the dependent variable. The independent variables were selected based on their reported effect on response rates in previous literature [8, 14, 22]. Furthermore, interactions between independent variables were checked and, when relevant, included in the model. Model assumptions for multicollinearity were checked by calculating the variance inflation factor (VIF) and goodness of fit was verified with a Hosmer Lemeshow test and model chi-squared test. A minimum sample size of 387 was required based on a power calculation for the primary outcome, which used the difference in response rates in previous research (effect size w = 0.2, α = 0.05, 1-β = 0.95).

All statistical analyses were performed in SPSS version 26 (Armonk, NY: IBM Corp). A *p*-value < 0.05 was considered statistically significant. Demographic information was available from a previous 2014 study, so there were no missing data for any demographic variables.

## Results

In total, 602 patients were approached, of which 45 (7%) refused participation, 170 (28%) did not respond, and 387 (64%) responded, as is shown in Fig. 1. Baseline characteristics of the patients in the three groups are shown in Table 1. As expected, the matching variables age and time elapsed since the last visit were equally distributed in all groups. The proportion of patients with a low level of education was higher in the mail group. Patients with a lower educational level, aged between 50 and 59 years or > 80 years, or > 5 years since the last visit were more often non-responders (Table 2).

**Table 1:** Baseline characteristics and response rates

	Stringent/intention to treat			As treated		
	Email (*N* = 202)	Hybrid (*N* = 204)	Mail (*N* = 196)	Email (*N* = 201)	Hybrid (*N* = 151)	Mail (*N* = 250)
**Sex (female)**	98 (49%)	107 (53%)	91 (46%)	97 (48%)	76 (50%)	123 (49%)
**Age**						
< 50 yrs.	11 (5%)	11 (5%)	9 (5%)	10 (5%)	11 (7%)	10 (4%)
50–59 yrs.	40 (20%)	40 (20%)	38 (20%)	39 (19%)	35 (23%)	44 (18%)
60–69 yrs.	62 (31%)	63 (31%)	61 (31%)	67 (33%)	53 (35%)	66 (26%)
70–79 yrs.	68 (34%)	70 (34%)	67 (34%)	60 (30%)	41 (27%)	104 (42%)
> 79 yrs.	21 (10%)	20 (10%)	21 (11%)	25 (12%)	11 (7%)	26 (10%)
**Education level**						
Low	55 (27%)	78 (38%)	84 (43%)	64 (32%)	48 (32%)	105 (42%)
Middle	59 (29%)	52 (26%)	51 (26%)	57 (28%)	42 (28%)	63 (25%)
High	88 (44%)	72 (35%)	60 (31%)	80 (40%)	60 (40%)	80 (32%)
**Time since last visit**						
< 5 yrs.	126 (62%)	125 (61%)	120 (61%)	122 (60%)	91 (60%)	160 (64%)
≥ 5 yrs.	76 (38%)	79 (39%)	76 (39%)	81 (40%)	60 (40%)	90 (36%)
**Response rate**						
Stringent	75 (37%)	77 (38%)	112 (57%)			
After 1st reminder	91 (45%)	119 (58%)	118 (60%)	85 (42%)	77 (51%)	166 (66%)
After telephone call	126 (62%)	136 (67%)	125 (64%)	120 (60%)	94 (62%)	173 (69%)
**Different delivery method**						
Email	–	4	20			
Mail	25	49	–			

Baseline characteristics of the three delivery methods for the stringent, intention to treat and as treated analysis are shown. The stringent response rate considered patients who switched delivery method as non-responders. In the intention to treat analysis patients are grouped in the delivery method category they were assigned to. In the as treated patients were grouped their actual delivery method category, since some patients had requested a different delivery method. Time since the last visit shows the years since the last consultation in the hospital

*yrs* years

**Table 2:** Non-responder analysis

	Responders	Non-responder	% responder
**N**	387	215	64%
**Sex**			
Female	187 (48%)	109 (51%)	63%
Male	200 (52%)	106 (49%)	65%
**Age**			
< 50 yrs.	20 (5%)	11 (5%)	64%
50–59 yrs.	66 (17%)	52 (24%)	56%
60–69 yrs.	125 (32%)	61 (28%)	67%
70–79 yrs.	143 (37%)	62 (29%)	70%
> 79 yrs.	33 (9%)	29 (14%)	53%
**Education level**			
Low	128 (33%)	89 (41%)	59%
Middle	109 (28%)	53 (25%)	67%
High	148 (38%)	72 (34%)	67%
**Time since the last visit**			
< 5 yrs.	254 (66%)	117 (54%)	69%
≥ 5 yrs.	133 (34%)	98 (46%)	58%

The demographics of overall responders (after first and second reminder) compared to non-responders. The percentages in the second and third columns reflect the percentage within the demographic group. The last column, % responder, reflects the percentage responders of each variable

Only 15 patients (2%) completed fewer than 80% of the total number of questions. Most incomplete responders in the email (*N* = 5) and hybrid (*N* = 6) groups seemed to have started the questionnaires and stopped at some point, without skipping items. In the mail group, incomplete responders (*N* = 4) skipped some questions. Because of the low number of incomplete responders, statistical analysis of differences in item or PRO level response rates or differences per PRO questionnaire could not be reliably performed.

Furthermore, 98 (16%) patients used the possibility to request a different delivery method: 74 (76%) preferred to receive a questionnaire by regular mail and 24 (24%) preferred to complete the questionnaire electronically (by email).

**Fig. 2:**
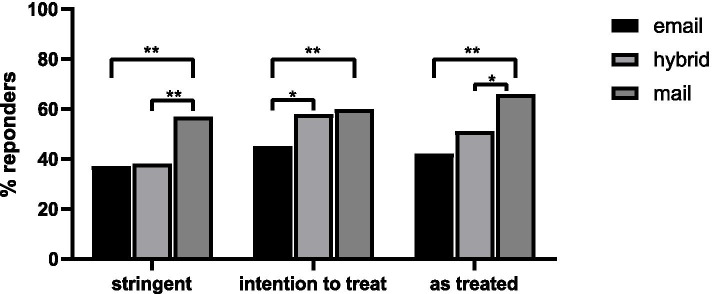
Response rates. The response rates of the different delivery methods are shown per analysis. In the stringent analysis, patients who requested a different delivery method are considered non-responders. In the intention to treat analysis all patients are analysed in their predefined group and in the as treated in their actual delivery method. * = χ^2^ *p*-value < 0.01. ** = χ^2^ *p*-value < 0.001

Figure 2 shows the results of the three performed analyses. In the stringent analysis, mail delivery resulted in statistically significantly better response rates compared to email and hybrid 57% versus 37 and 38%, respectively (χ^2^, *p* < 0.001). In the intention to treat analysis, when patients who switched delivery method were included, the response rates for patients allocated to delivery by email, hybrid, and regular mail were 45, 58 and 60%, respectively (χ^2^ *p* < 0.001).

The requests for a different delivery method resulted in a decrease in email (− 0.5%; *N* = -1) and hybrid delivery (− 26%; *N* = -53), and an increase in mail delivery (+ 28%; *N* = + 54), as is shown in Table 1. The response rate for the actual delivery method, shown in the as treated analysis, was 42% by email, 51% by hybrid, and 66% by mail (χ^2^, *p* < 0.001).

### Reminder by telephone

After the first reminder by either email or regular mail, 248 patients (41%) still did not respond and received a reminder by telephone call. Nearly half of these (*N* = 123) initial non-responders answered the telephone, of whom 48% (*N* = 59) did participate after this telephone call, 36% (*N* = 45) did not respond while they said to do so in the telephone call, and 15% (*N* = 19) declined participation. The demographics of these groups are shown in Table 3. The response rates in the intention to treat analysis raised to 62, 67, and 64% for email, hybrid and mail, respectively (χ^2^ *p* = 0.65). In the as treated analysis the final response rates were 60, 62 and 69%, respectively (χ^2^, *p* = 0.09).

**Table 3:** Effect of telephone call reminder

	Responders	Non-responders		
		Not answered	Non-responder despite promise	Refused to participate
**N (% of total)**	59 (24%)	125 (50%)	45 (18%)	19 (8%)
**Sex (female)**	32 (54%)	66 (53%)	23(50%)	11 (58%)
**Mean age (sd)**	65.9 (11.9)	65.9 (11.1)	63.8 (11.4)	71 (11.3)
**Education level**				
Low	24 (41%)	55 (44%)	13 (29%)	11 (58%)
Middle	20 (34%)	29 (23%)	16 (36%)	3 (16%)
High	15 (41%)	41 (33%)	16 (36%)	5 (26%)
**Time since the last visit**				
< 5 yrs.	40 (68%)	66 (53%)	23 (51%)	13 (68%)
≥ 5 yrs.	19 (32%)	59 (47%)	22 (49%)	6 (32%)

All non-responders (*N* = 248) were called 2 weeks after the first reminder. This table shows the demographics of this group. Half of the patients did not answer the telephone call. When patients did answer the telephone 59 out of 123 did participate, while 19 refused to participate. Another 45 patients promised to participate on the telephone but did not participate eventually

### Logistic regression

The results of the logistic regression are shown in Table 4. The stringent, intention to treat and as treated models met the model assumptions and goodness of fit tests. All models showed that the probability of responding was lower in the email delivery group. The hybrid delivery was also associated with a lower response rate in the stringent and the as treated models.

**Table 4:** Results of logistic regression

		Stringent		Intention to treat		As treated		
		OR	95%CI	OR	95% CI		OR	95% CI
**Delivery method**								
Email	*N* = 202	*0.24*	*(0.14–0.41)*	*0.32*	*(0.21–0.60)*	*N = 201*	*0.27*	*(0.16–0.45)*
Hybrid	*N* = 204	*0.34*	*(0.22–0.54)*	0.77	(0.49–1.21)	*N* = 151	*0.45*	*(0.28–0.71)*
Mail (reference)	*N* = 196	–		–		*N* = 250	–	
**Sex (female reference)**		1.16	(0.82–1.64)	1.08	(0.77–1.52)		1.11	(0.79–1.56)
**Age**								
< 50 yrs.		1.20	(0.47–3.08)	0.79	(0.32–1.96)		0.81	(0.32–2.03)
50–59 yrs.		1.54	(0.77–3.07)	1.16	(0.60–2.23)		1.13	(0.58–2.18)
60–69 yrs.		2.16	(1.13–4.01)	1.49	(0.81–2.72)		1.48	(0.80–2.74)
70–79 yrs.		1.43	(0.76–2.69)	1.69	(0.94–3.06)		1.49	(0.81–2.71)
> 79 yrs. (reference)		–		–			–	
**Education level**								
Low		*0.45*	*(0.29–0.69)*	*0.47*	*(0.31–0.72)*		*0.48*	*(0.31–0.73)*
Middle		0.87	(0.57–1.33)	0.75	(0.48–1.14)		0.76	(0.50–1.17)
High (reference)		–		–			–	
**Time since the last visit**								
< 5 yrs.		0.45	(0.18–1.12)	0.68	(0.28–1.72)		0.77	(0.31–1.87)
≥ 5 yrs. (reference)		–		–			–	
**Interaction term**								
time last visit x delivery method		*0.55*	*(0.36–0.84)*	0.67	(0.43–1.03)		0.72	(0.48–1.08)
**Model χ**^**2**^		< 0.001		< 0.001			< 0.001	
**Hosmer and Lemeshow**		0.43		0.51			0.18	

In all regression models, response rate was the dependent variable. The independent variables were delivery method, age, sex, education level, and time since the last visit. Also, the interaction term time since the last visit x delivery method was included; other interaction terms were not significant and therefore not included in the regression models. The χ^2^ and the Hosmer and Lemeshow show the *p*-values of the goodness of fit tests. The time since the last visit is the years since the last consultation in the hospital. Statistically significant effects are shown in italics

*OR* Odds ratio, *CI* confidence interval, *yrs* years

A low education level was a confounding factor in all models. Age and sex did not contribute to a lower or higher response rate, except for patients aged 60–69 years in the stringent model, who were more likely to respond.

**Fig. 3:**
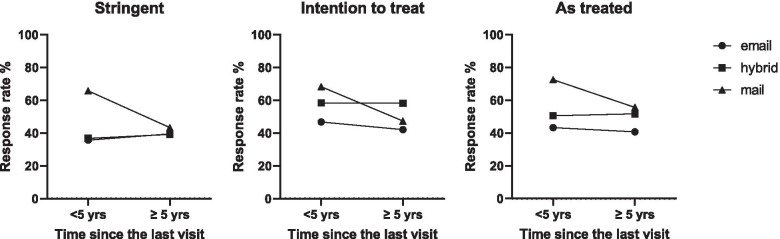
Interaction delivery method and time since the last visit. The time since the last visit affected the relation between delivery method and the response rate. In all analyses (stringent, intention to treat, as treated), response rate decreased with increasing time since last visit. This effect was not observed in the email and hybrid delivery groups

The interaction between the time since the last visit and delivery method was close to statistical significance in the intention to treat and as treated analyses. In the stringent analysis, this interaction was statistically significant, meaning that patients whose last visit to the hospital was less than 5 years ago tended to have different response rates per delivery method than patients whose last visit was longer ago. In the mail delivery group, the response rate decreased with increasing time since last visit. In the other groups, this effect was not observed, as is shown in Fig. 3. Other interactions (i.e. between age, sex, education level, and delivery method) were not statistically significant (lenient *p*-values of more than 0.2) and were not included in the models.

## Discussion

This study suggests that email delivery might result in a lower response rate compared to delivery by regular mail or hybrid delivery. Even when patients could choose their preferred delivery method, the response rate per email remained lower than mail or hybrid delivery.

The low response rate of email delivery is consistent with prior studies on patient response [12, 14]. This is somewhat surprising as one might expect increasing digital literacy in patients with the growing digitalization of the patient journey in hospitals today. Compared to other studies, we found smaller differences between the delivery methods, despite patients’ older average age in this study. An older population might be less familiar with the internet or email, but in The Netherlands, 87% of the elderly (> 65 years) have internet access, and 72% used email in 2019. In the subgroup of 65–75 years (which comprises approximately half our study population), these percentages are even higher: 95% internet access and 83% use of email [23].

Sex and education level could also act as confounding factors factors on response rate or interact with delivery method. For example, in healthcare-related research amongst patients, an effect of sex is not consistently observed [24, 25]. In this study too, sex did not seem to affect the response rate or vary the response rate by delivery method (i.e., no significant interaction with delivery method). The level of education did have a significant impact on response rates, as patients with a low level of education were less likely to be responders in this study (Table 4), which is consistent with a previous report [8]. However, the effect of the delivery method on response rate did not vary by education level. Finally, the time since last clinic visit appeared to affect the association between delivery method and response rate, as we observed a decreasing response rate with increasing time since last visit, but only in the mail delivery group (Fig. 3). This effect might be comparable to the effect of decreasing response rates with increasing follow-up periods, as reported in long-term follow-up studies, however it is unclear why this effect is only seen after mail delivery [26].

Although regular mail delivery had the highest response rate, there are some logistic disadvantages. To use the PROs, surveys on paper need to be digitized, which is time-consuming and error-prone. This is especially cumbersome when PROs are used in a clinical context, and feedback is expected during clinical consultation. In this light, the results of hybrid delivery are noteworthy since the response rate is close to regular mail delivery, but the PROs are completed and returned electronically. In practice, using a hybrid system could reduce the workload of digitizing PRO outcomes, with comparable response rates to surveys by mail.

In addition, a telephone call reminder can further increase response rates. In the current study, 48% of initial non-responders did respond after being reminded by a telephone call. However, the advantage of this higher response rate should be weighed against the time investment needed.

There are some inherent limitations to this study. First, it was impossible to perform a randomized trial because an email address was not available for all patients eligible for inclusion. Although the missing email addresses were caused by a different registration system in the hospital, we cannot be entirely sure that the differences between the groups are purely random. Second, the study participants were probably prone to participate in a research survey because they had already participated in a previous study in 2014. This committed population may therefore have increased response rates. Conversely, a decreased response rate may have been caused by a prolonged time interval between the survey and the last consultation, as was observed in a number of participants and was associated with a lower probability of responding in this study. Last, the PRO measures response rates found in this cross-sectional research setting may not be representative of PRO measures response rates in a clinical setting, in which PRO measures are typically collected close before or after a clinical consultation and serve a more direct clinical purpose. However, patient preferences with regard to the survey delivery method are probably equally applicable to both settings.

When using PRO measures, the response rate is an essential factor to consider. Various factors have been identified that influence the response rate, such as personally addressed invitations, shorter questionnaires, and financial incentives [7, 27, 28]. In the current study, all invitations were personally addressed, but no financial incentives or differences in questionnaire lengths were applied. In addition, we found that a reminder by letter and/or telephone call may be a particularly important factor in increasing the response rate of patients, which is in agreement with previous report on health studies [7]. In addition, this study suggests that two other factors are of importance in patients’ response rates: the initial delivery method and the ability to choose the desired delivery method.

## Conclusion

The effectiveness of the increasing use of PROs in healthcare stands or falls by patients completing and returning the questionnaires. This response rate can be influenced by several aspects, and the current study suggests that the route of survey delivery is an important factor. Regular mail delivery seems to perform better than email delivery in our study population but is more time-consuming, both in distribution, and in digitalization afterwards. Therefore, a hybrid delivery method in which patients receive a letter by regular mail with a code to access the survey digitally might be the best of both worlds.

## Data Availability

The dataset generated and analysed during the current study is available in the DANS/EASY repository: 10.17026/dans-xak-tvya

## Abbreviations

PRO: Patient Reported Outcome
HRQL: Health Related Quality of Life
SF-36: Short Form 36
PANQOL: Penn Acoustic Neuroma Quality-of-Life Scale
DHI: Dizziness handicap inventory
MOS-CFS: Medical Outcomes Study – Cognitive Functioning Scale
iPCQ: iMTA Productivity Costs Questionnaires
